# Modelling of Fluid Permeability at the Interface of the Metal-to-Metal Sealing Surface

**DOI:** 10.3390/ma17215194

**Published:** 2024-10-24

**Authors:** Przemysław Jaszak, Jan Oredsson, Rafał Grzejda

**Affiliations:** 1 Faculty of Mechanical and Power Engineering, Wroclaw University of Science Technology, Wybrzeze Stanislawa Wyspianskiego St. 27, 50-370 Wroclaw, Poland; 2 Pipeotech AS, Molandsveien 10, 4994 Akland, Norway; jan@pipeotech.com; 3 Faculty of Mechanical Engineering and Mechatronics, West Pomeranian University of Technology in Szczecin, 19 Piastow Ave., 70-310 Szczecin, Poland

**Keywords:** metal gasket, fluid permeability, contact pressure, fractal method, finite element method

## Abstract

This paper presents a method for modelling the permeability of fluid at the interface formed between flat parallel plates and the sharp-edged ridges of a metal gasket. This work was divided into three stages. In the first stage, numerical calculations simulating the deformation (compression of the gasket) were performed. The calculations were carried out using thermomechanical static analysis with commercial software. The purpose of these calculations was to determine the contact area of the gasket ridges with the plates, the deformation of the gasket ridges, and the reaction force resulting from the degree of compression of the gasket. In the second part of this work, analytical calculations were performed to estimate the tightness level. The analytical model proposed in this paper was based on Darcy’s equation, simulating fluid flow through a ring-shaped porous layer. The analytical model also took into account the shape of the roughness profile of the sealed surfaces. A mathematical Ausloos–Berman function based on fractal theory was used to represent it. In the last part of this work, experimental tests were carried out to determine the actual fluid permeability and thus verify the numerical and analytical calculations.

## 1. Introduction

Bolted joints are one of the most commonly used solutions for connecting flanges in piping systems [1,2,3,4]. This is particularly the case in the petrochemical, oil and gas, and chemical industries. The proper preparation of such joints can determine the tightness of piping systems. Meanwhile, the modelling of permeability at the interface of two materials (for example, a pair of pipe flanges) remains a topical issue, attracting the attention of a wide range of scientists and engineers involved in sealing the engineering of machine parts and technical equipment [5,6,7].

Maintaining leak-tightness at the interface between two materials, usually with different physical and chemical properties, poses certain challenges due to the interdisciplinary nature of the phenomena that occur in this area. These include thermomechanical phenomena (such as deformation and thermal expansion), phenomena associated with the flow of a fluid through a so-called porous bed, as well as aspects related to the structure and topography of the surface [8,9,10].

Recently, so-called metal gaskets have gained wide popularity in the sealing technology of pipelines and industrial fittings. They are characterised by high mechanical and thermal strength. Nevertheless, careful workmanship, both of the gasket itself and of the surface to be sealed, is required to ensure tightness at the metal-to-metal interface [11].

A key parameter affecting the permeability of metal gaskets is surface roughness. The influence of this parameter on the assessment of permeability and its mathematical modelling can be found in many papers. Zhang et al. [12] approached the modelling of permeability at the interface between two metallic surfaces using fractal theory. The mathematical model proposed by the authors reproduces the contact geometry of two metal surfaces, one rough and deformable and the other perfectly smooth and rigid. The level of permeability is the result of the gradual interpenetration of the two surfaces under compressive force, causing the pore cross-sectional area of the rough contact to gradually decrease. For the mathematical representation of the rough surface, the authors used the three-dimensional model proposed by Ausloos and Berman [13]. The final part of the study also modelled the fluid flow through an annular porous bed, replicating the operating conditions of a typical metal gasket. Experimental verification of the mathematical model described above is presented in [14]. The authors carried out tests on a test rig, the main components of which were sets of two circular plates with different contact surface roughness. The tests consisted of measuring the leakage (i.e., permeability) through the contact of these surfaces at different values of contact force and pressure of the fluid to be sealed. The final conclusions show that a very good agreement between the mathematical model proposed in [12] and the experiment was obtained.

Zheng et al. [15] addressed the effect of temperature on the thermal expansion of the interface and hence on the permeability of two metallic surfaces. The results of the mathematical modelling of the leakage at the interface between two surfaces are also described in [16], introducing the concept of the so-called flow regime, which distinguishes between bulk and diffusive flow through a porous bed. An analytical model of fluid flow through a porous interface using, among others, the Sierpinski fractal model was proposed in [17]. The results of work dealing with the mathematical modelling of the deformability of a rough surface under pressure and its direct effect on the geometry of a single pore are presented in [18,19,20], among others. A method for modelling leakage at the metal-to-metal interface was also proposed in [21], where the permeability of the sealed fluid was reproduced with high accuracy on the basis of an analytical model and numerical calculations (i.e., simulation of the deformation of two rough surfaces under pressure).

Analysis according to computational fluid dynamics (CFDs) [22] can also be used for modelling leakage. For example, this analysis was used by Zhang et al. [23] to model the flow at the interface of two deformable surfaces, taking into account the effect of non-uniform stress distribution due to external loading. Grine and Bouzid [24] proposed an analytical modelling of leakage through a porous bed, giving two models of fluid flow through a single pore, i.e., a capillary model and an annular model. The simulation results were verified experimentally, achieving very good agreement.

A typically experimental way of assessing permeability at the metal gasket–metal plate interface is presented in [25], in which quantitative results of leakage (i.e., permeability) induced by varying degrees of loading (compression of the plates) and pressure of the fluid being sealed are shown. An empirical model for assessing leakage in a flanged joint with a gasket is proposed in [26].

In contrast to the work shown above, this paper addresses the modelling of leakage on the unusual metal-to-metal interface geometry of metal plates with sharp-edged gaskets. The gasket analysed in this work is characterised by three concentric rings incised on the outer surfaces, which cause extremely high contact pressures even at low axial loads on the plates between which the gasket is fitted. Using the mathematical models proposed in the above-described studies, it is not possible to reproduce the leakage (permeability) effect in this case, because these models assume a contact geometry of two parallel surfaces which, under the influence of displacement (compression), interpenetrate each other and reduce the cross-sectional area of the rough surface. In the case of the proposed innovative gasket, one of the surfaces is not parallel but sharply edged, so the intensity of its penetration into the roughness profile of the sealed surface has a dominant effect on pore closure and thus permeability. This paper uses the well-known fractal description of a rough surface [13], but in the analytical model of the cooperation between the two surfaces, the change in pore geometry is described by the gradual immersion of the gasket ridge. The proposed model is a semi-empirical formulation, as some of the results needed for the analytical calculations were imported from numerical calculations. The model was verified experimentally by carrying out leakage and compression tests of the gasket under different loading conditions, i.e., the effects of temperature and load. It is worth adding that the tests and simulations of gasket compression do not fully reflect the real deformation of the gasket ridges that would occur in a real bolted flange joint. Considering this effect will be the subject of future research.

## 2. Materials and Methods

### 2.1. Object of the Research

**Figure 1:**
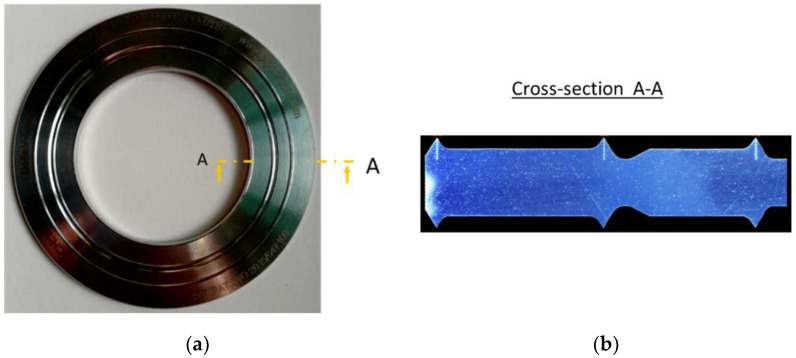
Design of a metal gasket of the DeltaV-Seal^TM^ type: (**a**) front view; (**b**) cross-section.

The object of this research was a metal gasket of the DeltaV-Seal^TM^ type [27,28]. Its characteristic feature is that the sealing surface is formed by sharp-edged rings, concentrically incised on the faces of the gasket, which form concentric contact surfaces during compression [29] when the tops of the ridges are flattened. The gasket design in the analysed variant has three sharp-edged rings located on the inner, middle, and outer diameters, respectively, as shown in Figure 1. The fundamental advantage of such a design over typical flat gaskets is that the sealing mechanism in this case relies partly on the elastic–plastic deformation of the ridges and their gradual penetration into the rough surfaces of the components to be sealed. Such gaskets are mainly used in bolted flange joints of pipelines and fittings [30]. They are characterised by very high tightness and mechanical and thermal strength.

**Figure 2:**
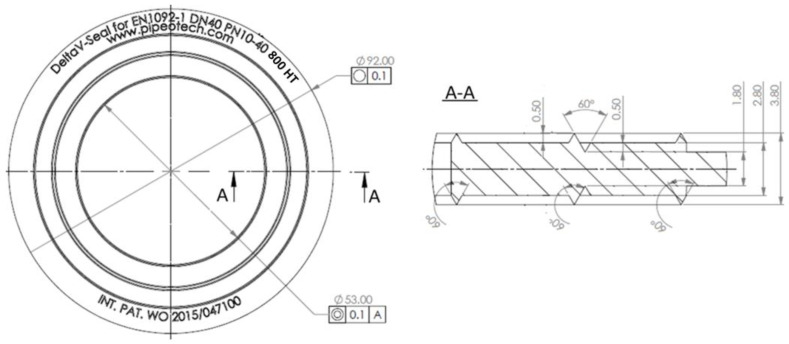
Characteristic dimensions of the gasket used in the tests.

In the case of the DeltaV-Seal^TM^, the sealed surface rests in three places across the width of the gasket, which increases the load-bearing capacity of the joint and, above all, prevents excessive rotation of the flanges during bolt tightening [31,32]. The material for the gasket can be S235 carbon steel, 316L or 304L acid-resistant steel, or 800HT high-temperature steel or other metallic materials. The standard dimensions of this type of gasket range from 1/2″ to 24″ or on the EN scale from DN 15 to DN 600 [33]. In this study, gaskets made of 800HT material and dimensions corresponding to DN40 PN40 [33] designation were used for testing. The characteristic dimensions of the gasket are shown in Figure 2.

### 2.2. Numerical Calculations

The primary objective of the numerical calculations was to determine the actual contact area, mainly of the inner ring (which is the main seal for internal containment), its deformation (compression ratio), and to identify the reaction force exerted on the top compression plate. The first of these two parameters (contact area of the inner ring and its deformation) provide the necessary data for analytical calculations of the degree of permeability (leakage). The third parameter allows for the leakage or deformation characteristics to be plotted as a function of the exerted gasket contact stress. The calculations were carried out in Ansys Workbench 19.0 (Ansys, Inc., Southpointe 2600 Ansys Drive, Cecil Township, PA 15317, USA) using static thermal analysis in conjunction with structural analysis [34].

#### 2.2.1. Computational Model

**Figure 3:**
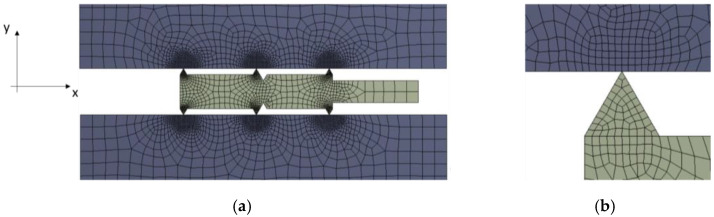
Axisymmetric finite element computational model: (**a**) general view; (**b**) mesh compaction.

Figure 3a shows an axisymmetric finite element computational model. This model represents a gasket placed between two parallel surfaces. These surfaces represent a section of the plates of the hydraulic press on which the experimental tests, presented later in this paper, were carried out. PLANE182 finite elements [35] with a second-order shape function were used to discretise the model. The basic edge dimension of the element was 1 mm. In the areas of expected stress increase (at the edges of the gasket and where it meets the plates), the design mesh was compacted to an element edge dimension of 0.05 mm (see Figure 3b). This was the optimum value, as a further reduction in the element dimension only slightly affected the value and stress distribution in the contact area.

The contact between the edges of the gasket and the press plates was modelled as frictional, with a friction coefficient of 0.3. Due to the high stiffness of the plates, relative to the edges of the gasket, an elastic model was chosen to represent their material properties. For the gasket material, a bilinear elastic–plastic model was used. The material data of 800HT were determined during the compression test at room and elevated temperature. The mechanical properties of both material models are shown in Table 1.

The numerical model was defined as static, with an active nonlinear material function. The equilibrium equation was formulated in this case as follows:

(1) $$K_{T}\Delta U={(K}_{0}+K_{d}+K_{\sigma })\Delta U=\Delta F$$

where ***K****_T_*—tangent stiffness matrix; ***K***_0_—small displacement stiffness matrix; ***K****_d_*—large displacement stiffness matrix; ***K****_σ_*—initial stress matrix dependent on the stress level; Δ***U***—generalised displacement vector; and Δ***F***—difference between applied force and resistance force (vector).

The individual stiffness matrices are as follows:

(2) $$K_{0}=\int_{V_{0}}^{-}B_{0}^{T}DB_{0}\;{dV}_{0}$$

(3) $$K_{d}=\int_{V_{0}}^{-}\left(B_{0}^{T}DB_{L}+B_{L}^{T}DB_{0}+B_{L}^{T}DB_{L}\right)\;{dV}_{0}$$

(4) $$K_{\sigma }=\int_{V_{0}}^{-}B_{N}^{T}SB_{N}\;{dV}_{0}$$

where ***B***_0_—linear strain–displacement transformation matrix; ***B****_L_*—linear strain–displacement transformation matrix, which depends on the displacement; ***B****_N_*—nonlinear strain–displacement transformation matrix; ***D***—elasticity matrix; and ***S***—Piola–Kirchhoff second stress tensor.

Equation (1) was solved using the Newton–Raphson incremental method.

#### 2.2.2. Boundary Conditions

**Figure 4:**
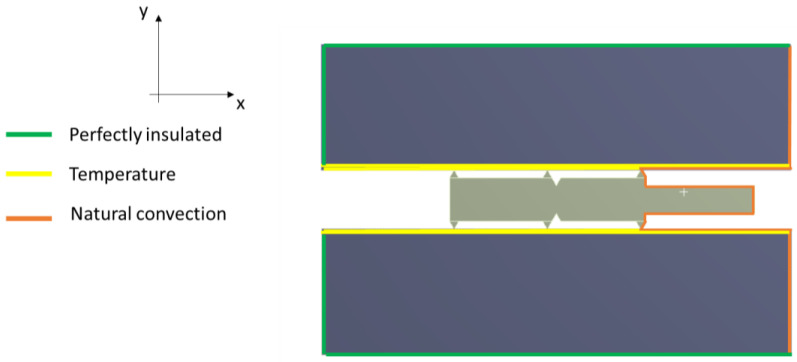
Thermal boundary conditions of the computational model.

The first loading case for the model was to determine the loads used in the thermal analysis. For this purpose, the leading edges of the plates were loaded with temperature, depending on the analysis case. These were assumed to be 20 °C, 200 °C, and 400 °C, respectively. At the outer edges of the gasket and the outer edges of the plates, natural convection conditions were introduced, with a convection coefficient of 5 W/(m^2^·°C). Thermal insulation conditions were applied to the remaining edges of the plates. The thermal conditions considered are shown in Figure 4.

**Figure 5:**
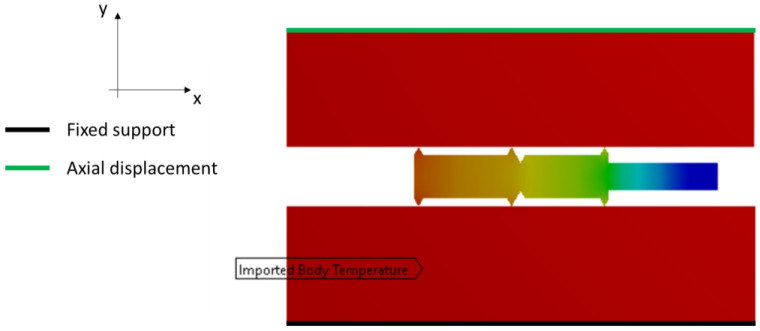
Boundary conditions applied in the structural analysis.

The second loading case (used in the structural analysis) was first to transfer the temperature distribution field obtained from the thermal analysis calculations and then to define the boundary conditions corresponding to the gasket compression simulation. The displacement condition was applied to the upper edge of the top plate, while the lower edge of the bottom plate was fixed, taking away all degrees of freedom. The maximum displacement of the top plate depended on the temperature considered and ranged from 0.4 mm to 0.5 mm. The given boundary conditions used in the structural analysis are shown in Figure 5.

#### 2.2.3. Reading the Results

**Figure 6:**
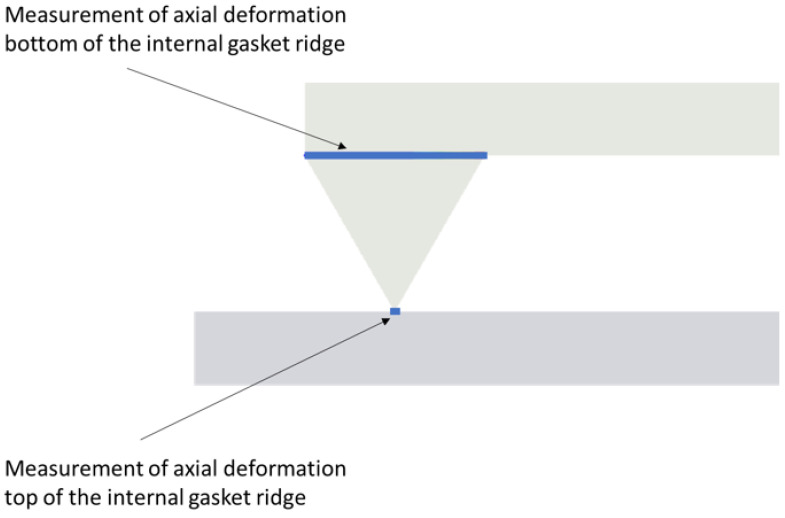
Measuring paths for outer ridge deformation.

To determine the force inducing the compression of the gasket, a reaction force measurement resulting from a given displacement was introduced to the top edge of the upper plate. Relating the reaction force of the top plate to its displacement allowed for the determination of the force-displacement characteristics and the contact stress characteristics of the gasket as a function of displacement. In order to determine the precise deformation of a single sealing ridge, a displacement measurement was applied to each ridge at its base and top (see Figure 6).

**Figure 7:**
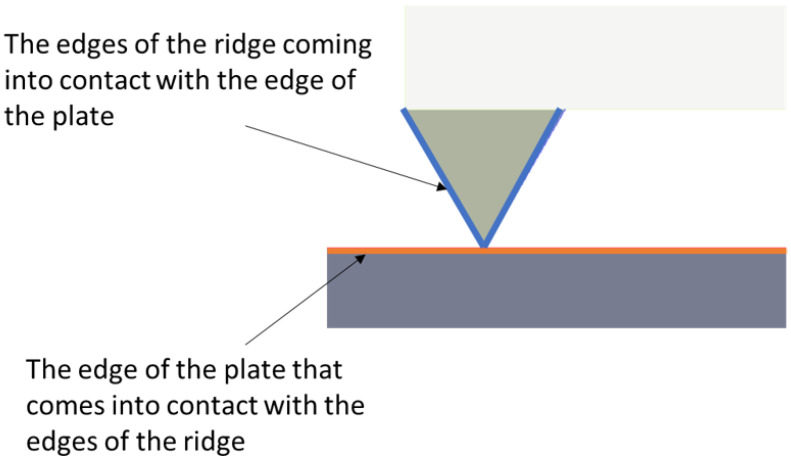
Measuring paths for the contact between the outer ridge of the gasket and the surface of the lower plate.

It was also a necessary measurement to determine the actual internal contact area of the gasket ridge as a function of its displacement. To this end, a measurement of the contact between the surface of the ridge and the surface of the lower plate was introduced (see Figure 7).

### 2.3. Analytical Model

The following well-known equation was adopted to model the permeability at the interface of porous metal surfaces *K_V_*:

(5) $$K_{V}=\frac{\pi D_{f}\lambda_{max}^{4}}{128\tau \left(4-D_{f}\right)S}$$

where *D_f_*—fractal dimension of tortuous capillaries; λ*_max_*—maximum pore diameter; τ—tortuosity of the capillaries; and *S*—cross-section of the flow.

Equation (5) describes the permeability through a porous bed formed by the interface between two rough surfaces. The flow takes place along dislocated capillaries with a maximum pore diameter, which is characterised by the fractal dimension *D_f_*. To determine the parameters of Equation (5), it is necessary to know the characteristic dimensions of the roughness profile of the mating surfaces.

Accordingly, the first step in developing the analytical model was to mathematically represent the roughness profile of the sealed surfaces (in this case, the surfaces of the hydraulic press plates). For the mathematical representation of this profile, the Ausloos–Berman model [13] mentioned in the introduction was used in the following form:

(6) $$z\left(x,y\right)=L{\left(\frac{G}{L}\right)}^{D-2}{\left(\frac{ln\gamma }{M}\right)}^{\frac{1}{2}}\sum_{m=1}^{M}\sum_{n=n1}^{n_{max}}\gamma^{\left(D-3\right)n}\left\{cos\varphi_{a}\;\;\;\;\;\;\;\;\;\;\;\;\;\;\;\;\;-cos\left[\frac{2\pi \gamma^{n}{\left(x^{2}+y^{2}\right)}^{\frac{1}{2}}}{L}cos\left(artan\left(\frac{y}{x}\right)-\frac{\pi m}{M}\right)+\varphi_{a}\right]\right\}$$

where *z*—height of the surface profile; *L*—sample length; *G*—fractal roughness constant; *D*—fractal dimension (in the domain of 2 < *D* < 3); γ—frequency spectrum (usually γ = 1.5); $\varphi_{a}$—random number in the range between 0 and 2π; *n*—frequency index of asperities; *M*—number of superposed ridges; *n*—frequency index of asperities; *x*, *y*—coordinates (directions) of the surface profile.

As shown in [21] for the representation of face surfaces with concentric structure (i.e., those obtained by face turning machining, in which the roughness in the radial direction significantly exceeds the roughness in the circumferential direction), the Ausloos–Berman model can be simplified to a two-dimensional form, in which the parameter *M* = 1 is as follows:

(7) $$z\left(x\right)=L{\left(\frac{G}{L}\right)}^{D-2}{\left(ln\gamma \right)}^{\frac{1}{2}}{\;\;l}^{\left(D-3\right)n}\left\{cos\varphi_{a}-cos\left[\varphi_{a}-\frac{2\pi \gamma x}{L}\right]\right\}$$

**Figure 8:**
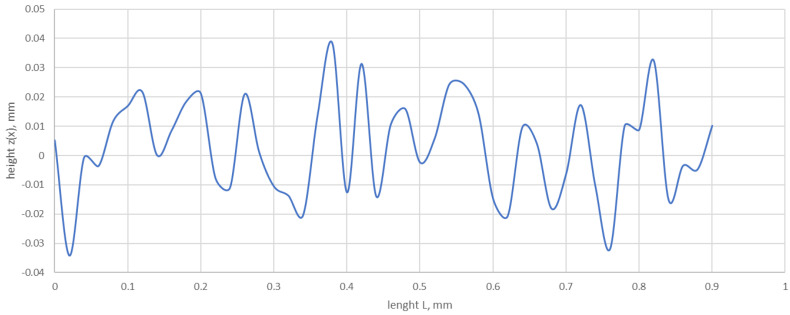
Roughness profile modelled by Equation (7) for *D* = 2.125, *G* = 1.87 × 10^−8^, *L* = 0.9 mm, and *M* = 1, γ = 1.5.

An example of a roughness profile mapped by Equation (7) is shown in Figure 8.

Equation (7) also makes it possible to model a single, characteristic roughness peak, i.e., one that appears most frequently along the length of the determined roughness profile. The single peak profile can then be described by a relation in which the length of the measurement segment *L* is equal to the length of the single peak base *l*:

(8) $$z\left(x\right)=G^{D-2}{\left(ln\gamma \right)}^{\frac{1}{2}}{\;\;l}^{\left(D-3\right)}\left\{cos\varphi_{a}-cos\left[\varphi_{a}-\frac{2\pi \gamma x}{l}\right]\right\};\;\left(0<x<l\right)$$

**Figure 9:**
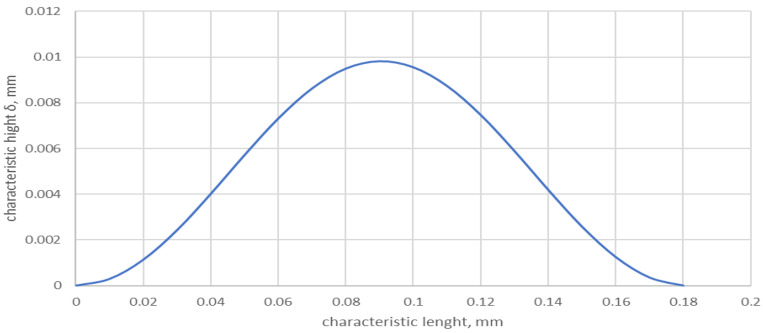
Example profile of a single roughness peak determined by Equation (4) for *l* = 0.18 mm, γ = 1.5, *D* = 2.1, and *G* = 1.9 × 10^−8^.

An example of a single peak profile is shown in Figure 9.

**Figure 10:**
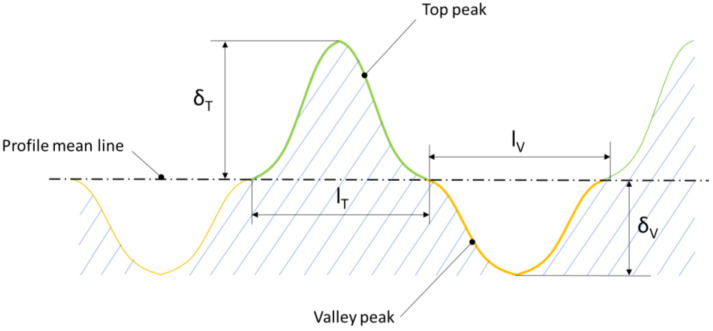
Basic geometrical parameters of the roughness profile.

It should be noted, however, that the term single peak refers to both the top and valley of the roughness profile. The boundary of their separation is the mean line of the profile, as presented in Figure 10.

The subscripts ‘*T*’ and ‘*V*’ used for the parameters shown in Figure 10 denote the top and valley, respectively, of a single roughness peak. The description of the profile of a single peak and valley, among other things, allows for their height, cross-sectional area, and perimeter to be determined, i.e., the necessary parameters for determining the diameter of the pore capillary, which is one of the dominant parameters that influence the level of permeability calculated by Equation (5).

When *x* = *l*/2 is inserted into Equation (8), the formula for the height of a single roughness peak δ is obtained:

(9) $$\delta =z\left(\frac{l}{2}\right)=2G^{D-2}{\left(ln\gamma \right)}^{1/2}l^{\left(3-D\right)};\;0<x<l$$

On the other hand, integrating Equation (8) gives the formula for the cross-sectional area of the characteristic peak (top or valley):

(10) $$A(x)=\int_{0}^{l}G^{D-2}{\left(ln\gamma \right)}^{1/2}l^{3-D}\left[1-cos\left(\frac{2\pi x}{l}\right)\right]dx=G^{D-2}{\left(ln\gamma \right)}^{1/2}l^{4-D};\;0<x<l$$

In turn, the perimeter of a single peak is received by the following operation:

(11) $$P(x)=\int_{0}^{l}\sqrt{1+{\left(2G^{D-2}{\left(ln\gamma \right)}^{1/2}l^{\left(3-D\right)}\right)}^{2}}dx;\;0<x<l\;$$

**Figure 11:**
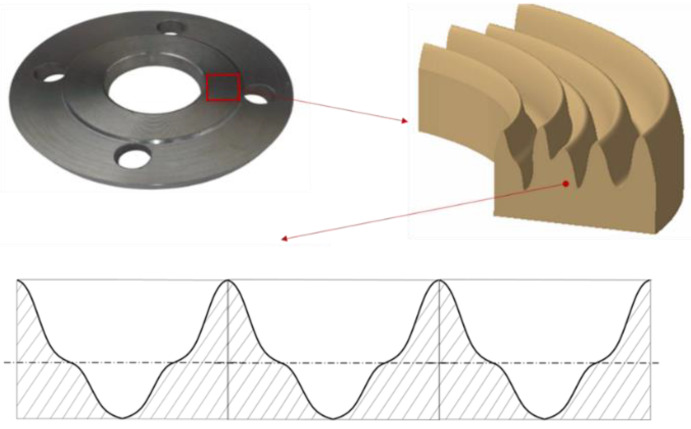
Example of replacing the actual roughness profile with a regular model profile.

By replacing the actual shape of the surface roughness profile of a face-turned surface with a model profile in which a single peak of top and valley roughness runs in a regular manner along the entire length of the roughness profile (Figure 11), its geometry can be determined using Equations (9)–(11). This geometry is necessary to determine the cross-sectional area of the pore, i.e., the area that is formed between the adjacent top and valley profiles.

**Figure 12:**
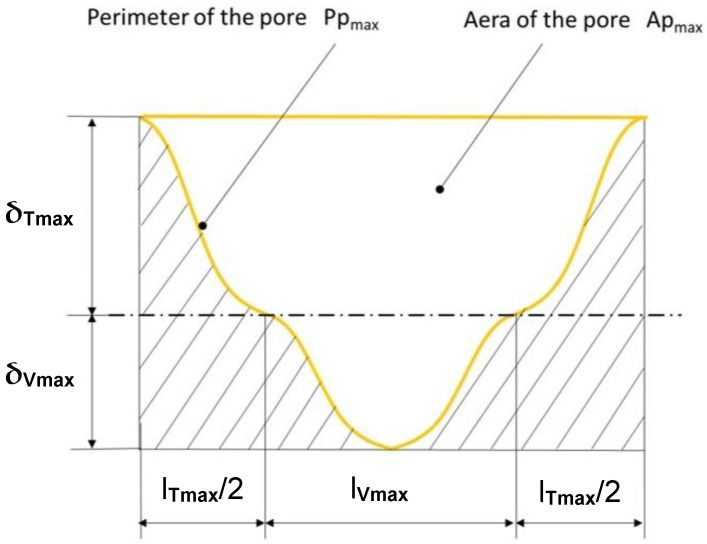
Basic parameters describing the shape of a single modelled section of the roughness profile.

The basic geometrical parameters describing the shape of a single modelled section of the roughness profile are shown in Figure 12.

As shown in [12], the dominant parameter affecting permeability is the cross-sectional area of the pore. This is the cross-sectional area formed between adjacent peaks of tops and valleys.

Based on Figure 12, the surface area of a pore of maximum dimension can be calculated from the following relationship:

(12) $$A_{Pmax}=\left(\delta_{Tmax}+\delta_{Vmax}\right)\cdot \left(l_{Tmax}+l_{Vmax}\right)-A_{Tmax}\;\;\;\;\;\;\;\;\;\;\;-{{((\delta }_{Vmax}\cdot l_{Vmax})-A}_{Vmax})-l_{Tmax}\cdot \delta_{Vmax}$$

where *A_Tmax_*, *A_Vmax_*—maximum cross-sectional areas of the top and the valley, respectively, calculated by Equation (10); *l_Tmax_*, *l_Vmax_*—base lengths of the maximum top peak and valley peak read from the actual roughness profile of the surface to be sealed, respectively; and δ*_Tmax_*, δ*_Vmax_*—maximum height of the top peak and valley peak, respectively, calculated using Equation (9) after half the base width of the top or valley was substituted.

The perimeter of the pore, on the other hand, can be counted from the following relationship:

(13) $$P_{Pmax}=P_{Tmax}+P_{Vmax}+l_{Tmax}+l_{Vmax}$$

where *P_Tmax_*, *P_Vmax_*—perimeters of the maximum top peak and valley peak, respectively.

**Figure 13:**
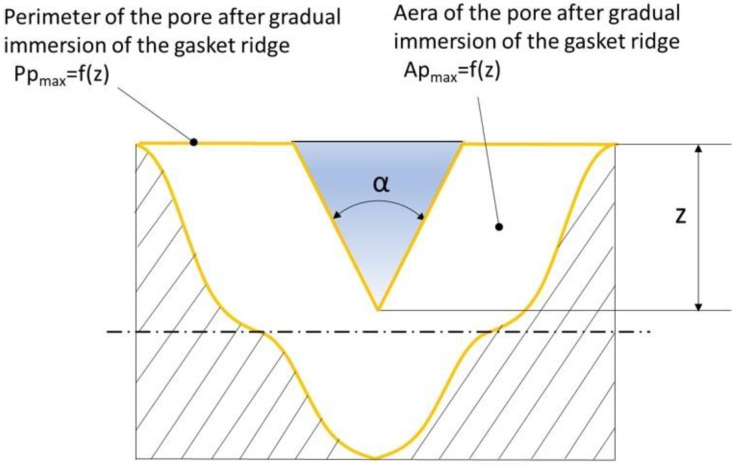
Cross-section of a pore after the sealing ridge was immersed in it.

In the case of gradual penetration of the sealing ridge into the pore section, Equations (12) and (13) reduce to relations (14) and (15), respectively. This is graphically illustrated in Figure 13.

(14) $$A_{Pmax}\left(z\right)=A_{Pmax}-z^{2}\cdot tg\left(\frac{\alpha }{2}\right)$$

(15) $$P_{Pmax}\left(z\right)=P_{Tmax}+P_{Vmax}+l_{Tmax}+l_{Vmax}-2\cdot z\cdot tg\left(\frac{\alpha }{2}\right)+2\cdot \frac{z}{cos\left(\frac{\alpha }{2}\right)}$$

By knowing the geometry of the pore cross-section, including the sealing ridge that fills it, the concept of the porosity level of the model section of the roughness profile as a function of ridge immersion ε(*z*) can be introduced:

(16) $$\varepsilon (z)=\frac{A_{Pmax}(z)}{\left(\delta_{Tmax}+\delta_{Vmax}\right)\cdot \left(l_{Tmax}+l_{Vmax}\right)}$$

**Figure 14:**
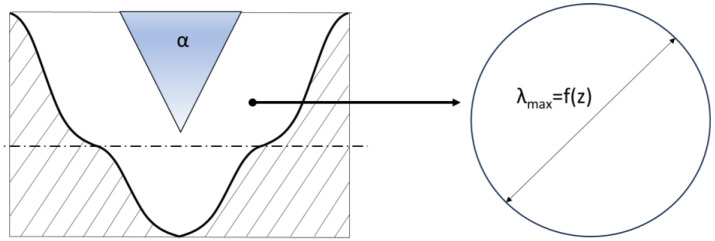
Graphical representation of the definition of equivalent pore diameter.

The diameter of the pore, in turn, can be determined from the analogy of the hydraulic diameter, in which the non-circular cross-sectional area is replaced by an equivalent circular cross-section, and its diameter is determined from Equation (17). A graphical interpretation of the calculation of the equivalent pore diameter is shown in Figure 14.

(17) $$\lambda_{Pmax}(z)=\frac{4\cdot A_{Pmax}(z)}{P_{Pmax}(z)}$$

The diameter of the pore and the degree of porosity allow for the determination of further parameters, which are the fractal dimension of tortuous capillaries *D_f_* and the tortuosity of the capillaries τ:

(18) $$D_{f}=2-\frac{\ln(\varepsilon )}{ln\left(\frac{\lambda_{min}}{\lambda_{max}}\right)}$$

(19) $$\tau =\frac{1}{2}\left[1+\frac{1}{2}\sqrt{1-\varepsilon }+\sqrt{1-\varepsilon }\frac{\sqrt{{\left(\frac{1}{\sqrt{1-\varepsilon }-1}\right)}^{2}+\frac{1}{4}}}{1-\sqrt{1-\varepsilon }}\right]$$

By analysing Equations (16) and (17), it can be seen that with the degree of compression, the sealing ridge gradually fills the pore cross-section, leading to a reduction in the degree of contact porosity and the pore diameter.

The value of the cavity of the sealing ridge is related to its direct deformation, a value calculated from the numerical calculations presented in Section 2.2.

By calculating the contact permeability, the leakage at the interface with the inner sealing ridge can be calculated in turn, using Darcy’s equation [38,39], which describes leakage through a porous bed. In the case of a cylinder-shaped bed, the formula for determining leakage (by mass related to the average diameter of the cylinder) takes the following form:

(20) $$Q_{m}=\frac{2\pi K_{V}h\rho \left(p_{0}-p_{t}\right)}{\eta ln\left(\frac{r_{o}}{r_{i}}\right)\cdot r_{AV}}\cdot \rho$$

where *K_V_*—permeability; *h*—height of the porous structure; *ρ*—fluid density; *p*_0_—pressure inside of the cylinder; *p_t_*—pressure outside of the cylinder; *η*—dynamic viscosity; *r_o_*—outer radius of the cylinder; *r_i_*—inner radius of the cylinder; and *r_AV_*—average radius of the cylinder.

**Figure 15:**
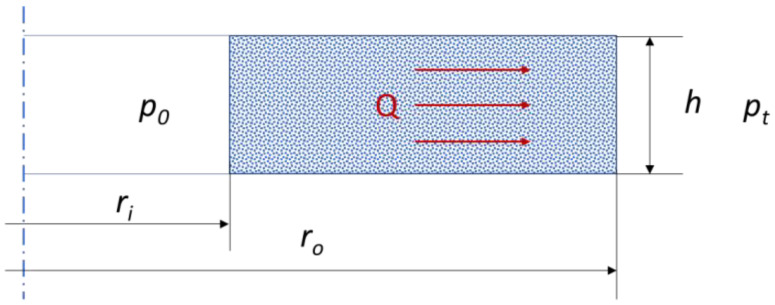
Graphical interpretation of a flow model through a porous bed in a cylindrical shape.

A graphical interpretation of Equation (20) is shown in Figure 15.

**Figure 16:**
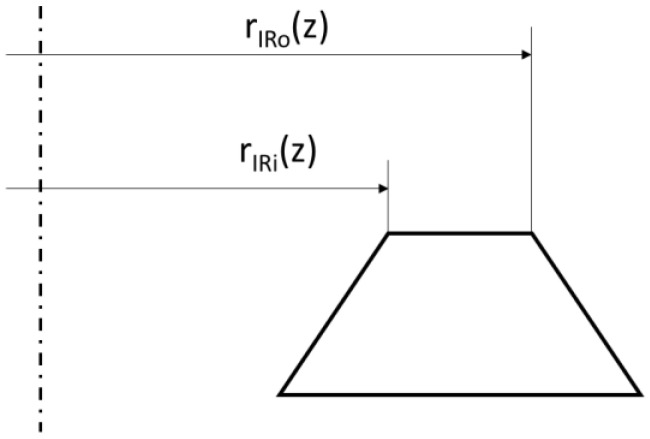
Graphical interpretation of deformed sealing ring.

It should be noted that the radii *r_i_* and *r_o_*, appearing in Equation (20), denoting the inner radius (*r_IRi_*(*z*)) and outer radius (*r_IRo_*(*z*)) of the sealing ridge, will vary with the degree of compression of the ridge, as shown in Figure 16. This is due to the contact width of the sealing ridge increasing with its axial load.

The contact width of the inner sealing ridge as a function of its deformation is calculated from the following relationship:

(21) $$b\left(z\right)=\frac{A_{IR}(z)}{2\pi r_{IRm}}$$

where *A_IR_*(*z*)—contact area of the inner sealing ridge as a function of the deformation *z*, obtained from numerical calculations; *r_IRm_*—mean radius of contact surface of inner sealing ridge.

The inner radius is calculated from the following formula:

(22) $$r_{IRi}(z)=r_{IRm}-b(z)$$

And the outer radius is calculated from the following equation:

(23) $$r_{IRo}(z)=r_{IRm}+b(z)$$

where *r_IRi_*(*z*), *r_IRo_*(*z*)—inner and outer radii of the contact surface of the outer sealing ridge.

### 2.4. Experimental Research

Experimental research was carried out on the samples that constituted the test subject described in Section 2.1. The first objective was to determine the characteristics describing the stiffness of the gaskets at a given temperature, on the basis of which the numerical model was verified. The second objective was to determine the gasket tightness as a function of the pressure exerted on the gasket, on the basis of which the analytical model was verified.

#### 2.4.1. Test Stand

**Figure 17:**
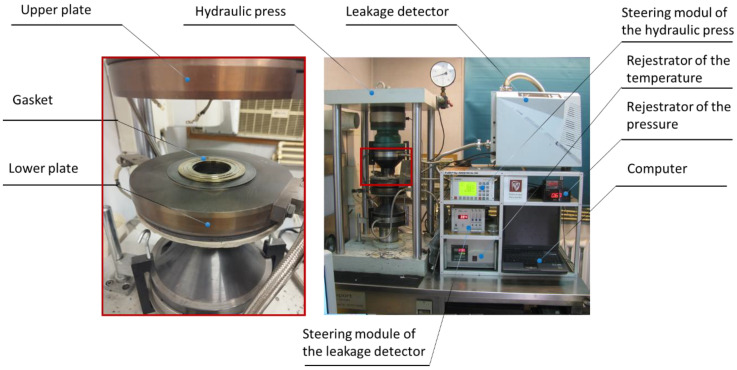
Test stand for measuring the tightness and mechanical properties of gaskets.

Figure 17 shows the test stand on which the experimental research was carried out. Its main component is a computer-controlled hydraulic press.

The press consists of a stationary lower plate and a sliding upper plate. A displacement sensor system is installed between the plates to record the deformation of the gasket under test. A force measurement sensor is used in the top plate. Both plates can be heated to a maximum temperature of 750 °C. A mass spectrometric helium detector was used to measure leakage. Helium was fed into the gasket from a cylinder connected by a line to the lower plate. A pressure gauge was installed in the path of this line to assess the pressure of the sealed medium. All measurement data were recorded in real time and archived in computer memory.

#### 2.4.2. Procedure for Determining the Compression Characteristics

The test procedure is based on EN 13555 [40,41], which strictly specifies the test plan for gaskets. It assumes a gradual compression of the gasket to given pressure levels and its partial unloading. This procedure allows for the plotting of so-called compression characteristics, which express the degree of compression of the gasket (change in thickness) as a function of the contact pressure exerted on its surface. In this test plan, gaskets were compressed at 20 °C, 200 °C, and 400 °C and at a maximum contact pressure of 700 MPa. The average contact pressure exerted on the gasket was calculated based on the force exerted by the hydraulic press relative to the fixed contact area of the gasket (referenced to the base of the ridges). The total contact area at the base of the three ridges in this case was 357.5 mm^2^. The results of the measurements are presented in Section 3.

#### 2.4.3. Procedure for Determining the Leakage Characteristics

This test procedure is also based on EN 13555 [40,41]. In this case, the tested gasket was compressed to a predetermined pressure level and unloaded. At each predetermined pressure point, a measurement of the leakage of helium gas sealed inside the gasket was taken. The pressure at which the leakage was measured equalled 40 bar. Tests were carried out at 20 °C, 200 °C, and 400 °C. The results of the tests are leakage characteristics, i.e., relationships defining the leakage level (related to the average gasket diameter) as a function of the contact pressure exerted on the gasket surface. The results of the measurements are presented in Section 3.

#### 2.4.4. Measurement of the Roughness Profile of Sealed Surfaces

The experimental study was complemented by the measurement of the roughness profile of the compression plates of the hydraulic press. The measurements were made using the contact method with an SRT-6600 HT profilometer (HUATEC Group Corporation, 7th Floor, Chengyuan Building B, the Mid. Road of Jiancaicheng Haidian Dist. Beijing, China), for which the length of the measuring section was 4.5 mm and the maximum measured depth was equal to 20 μm. The roughness profile parameters of the sealed surfaces defining the maximum ridge (its height and base width) were the basic data for determining the geometric parameters describing the analytical leakage model.

## 3. Results

### 3.1. Numerical Calculations

**Figure 18:**
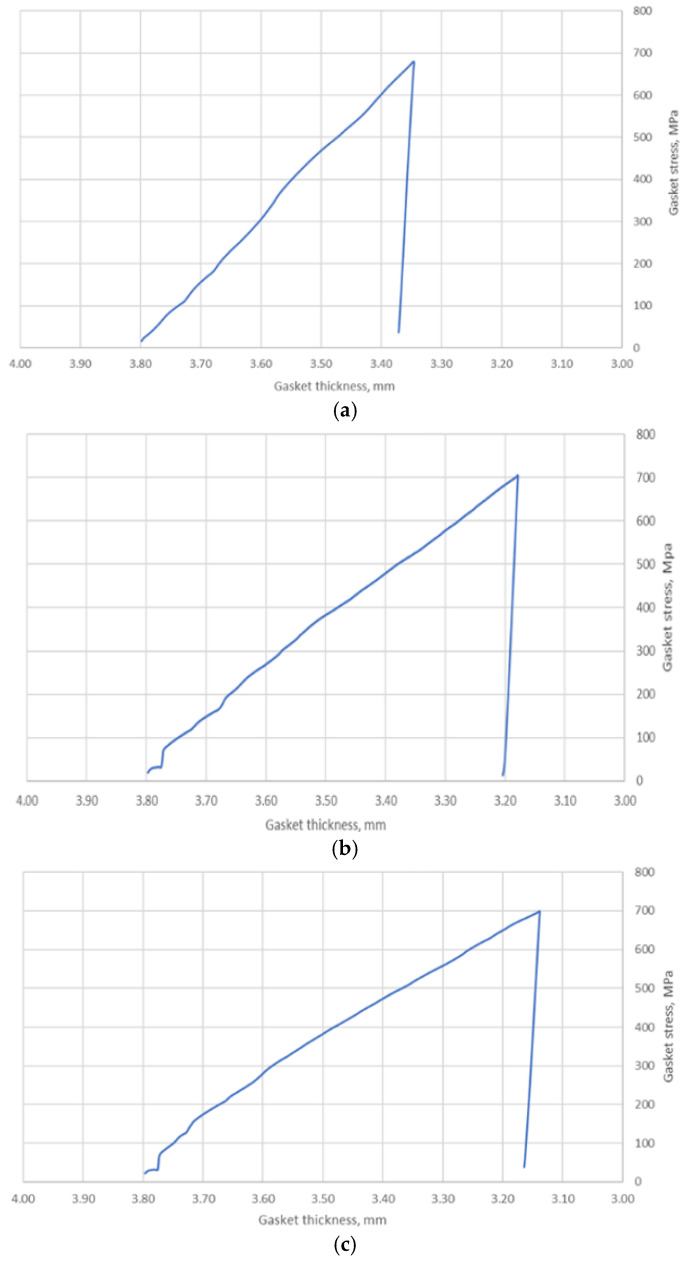
Compression characteristics of the gasket numerically determined at (**a**) 20 °C; (**b**) 200 °C; (**c**) 400 °C.

Figure 18 shows the compression characteristics of the gasket. These show the dependence of the contact pressure exerted on the gasket surface as a function of the change in gasket thickness. As in the experimental studies, the pressure was calculated here as the reaction force exerted on the top plate, related to the area resulting from the sum of the areas of the base of the three ridges.

**Figure 19:**
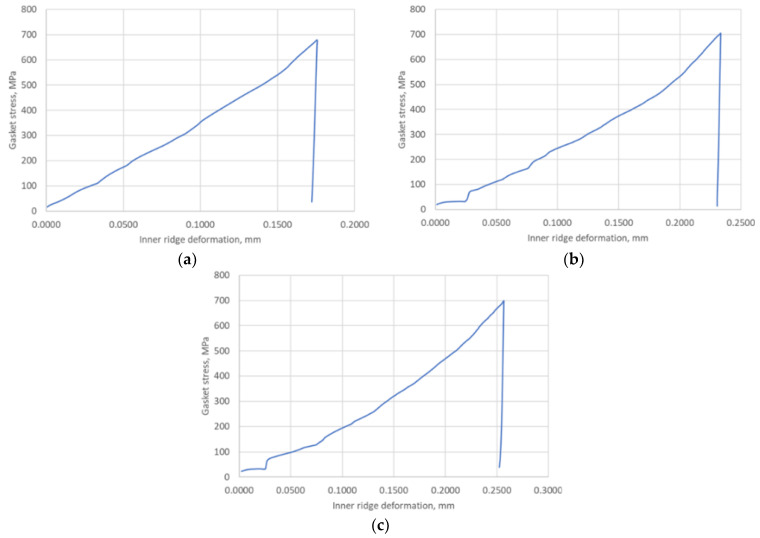
Deformation course of the inner sealing ridge under contact pressure at (**a**) 20 °C; (**b**) 200 °C; (**c**) 400 °C.

With a maximum displacement of the plate of 0.5 mm, the maximum pressure was approximately 700 MPa. The important parameter measured in the simulation, from the point of view of the analytical calculation, was the compression of a single ridge. For the purposes of the analytical model, the only and sufficient measurement was the plotting of this parameter for the inner ridge of the gasket. The course of its change at the three temperatures as a function of pressure is shown in Figure 19.

**Figure 20:**
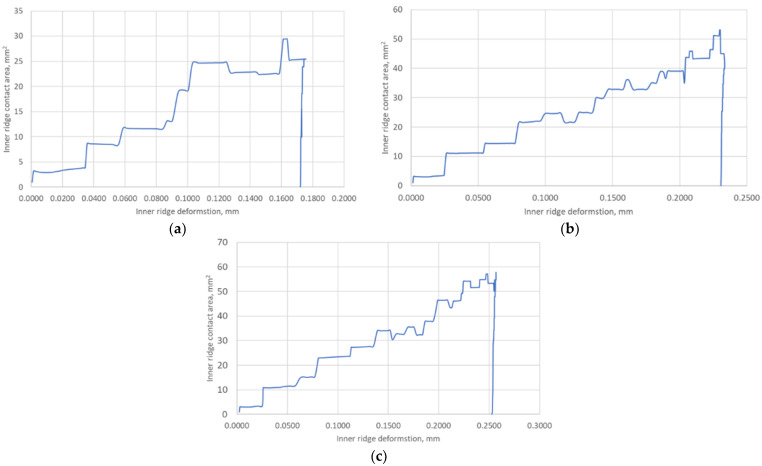
Course of the contact surface of the inner sealing ridge as a function of its deformation at (**a**) 20 °C; (**b**) 200 °C; (**c**) 400 °C.

The last of the important parameters needed for further analytical calculations was the change in the internal contact area of the ridge as a function of its compression to determine the actual contact width of the ridge (21). The course of the change in this parameter is shown in Figure 20.

As can be seen from the above data, a single ridge deforms from 0.175% to 0.255% depending on the temperature and the set maximum contact pressure, and the actual contact area varies from about 30 mm^2^ to about 60 mm^2^.

### 3.2. Results of Experimental Measurements

#### 3.2.1. Compression Characteristics

**Figure 21:**
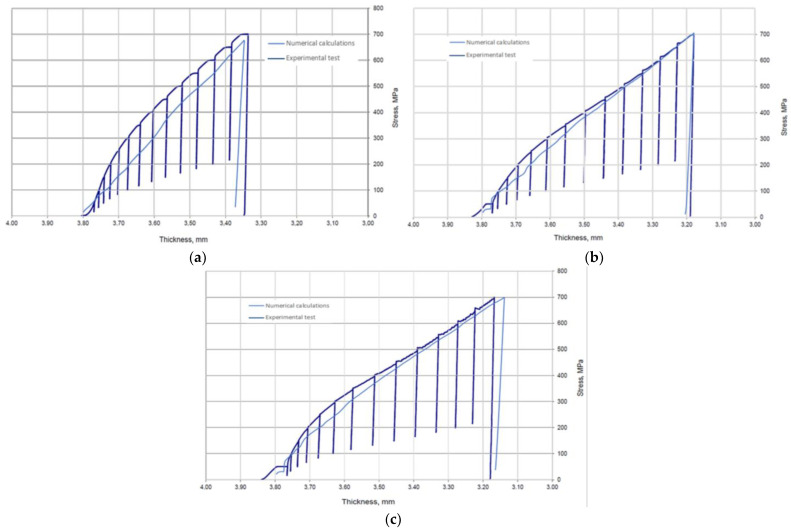
Actual compression characteristics of the gasket against numerically determined characteristics at (**a**) 20 °C; (**b**) 200 °C; (**c**) 400 °C.

Figure 21 shows the compression characteristics of the gaskets against the characteristics obtained from the numerical calculations. It can be seen that as the temperature increases, the experimental characteristics approach those determined numerically. The deviation of the numerically calculated characteristics from the actual characteristics is most likely due to errors in the thickness tolerances of the gaskets (individual ridges) resulting from inaccuracies in the turning treatment. This is evidenced by the fact that, as the compression of the gasket increases, the numerically determined characteristic approaches the experimental characteristic, which is the result of the gradual adaptation of the sealing ridges to the compression plates of the hydraulic press.

#### 3.2.2. Leakage Characteristics

**Figure 22:**
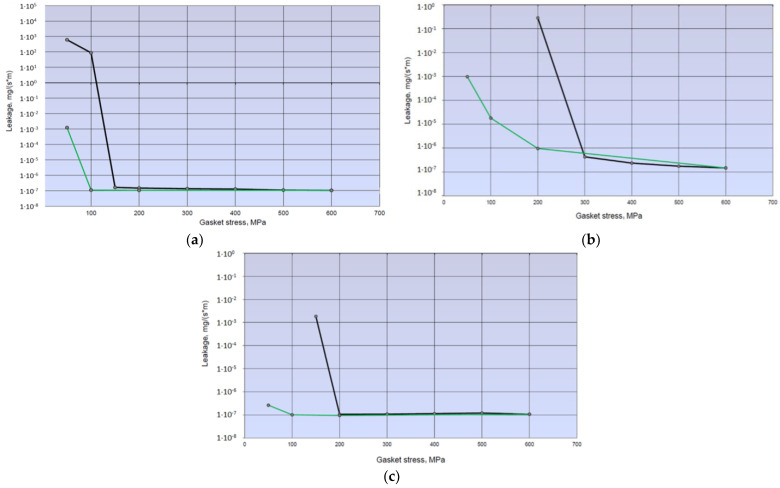
Leakage characteristics determined at (**a**) 20 °C; (**b**) 200 °C; (**c**) 400 °C (The black line indicates the experimental loading curve and the green line indicates the experimental unloading curve.)

Figure 22 shows the leakage characteristics that were obtained over a range of contact stress, from 600 MPa to 50 MPa.

The minimum leakage level, irrespective of the test temperature, oscillates between 1 × 10^−7^·mg/(m·s). What is noteworthy is the course of the characteristics in the area approaching the minimum leakage limit. Before this is reached, the leakage–contact pressure curve is very steep. A small increase in pressure results in a significant increase in leakage, even by several orders of magnitude. Once the minimum value is reached, the leakage already remains almost constant, regardless of the increase in contact pressure. When unloading, the leakage–pressure relationship curve (in the pressure range from 600 MPa to 200 MPa) deviates slightly from the load curve. A further decrease in contact stress shifts the limiting minimum leakage to the side of the lower stress values, which is due to the formation of hysteresis. This is mainly due to the plastic deformation of both materials (gasket and press plates). The plastic deformation of the gasket ridges has a particularly significant influence on this behaviour. The contact width of the ridge increases after compression and deforms permanently. This is particularly true of the contact width of the inner ring as it determines the gasket. The other two rings act more as secondary gaskets.

#### 3.2.3. Roughness Profile

**Figure 23:**
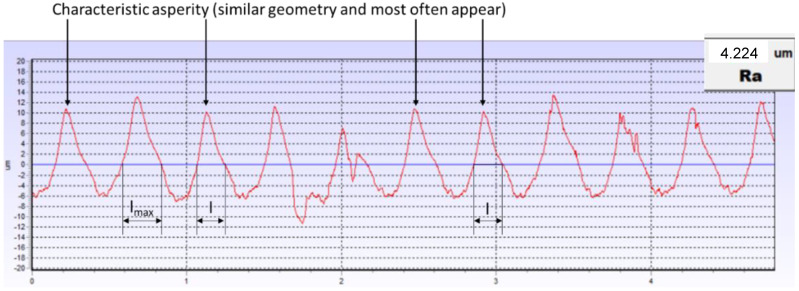
Roughness profile of the lower plate measured in the contact area with the tested gasket.

Figure 23 shows the roughness profile of the lower plate, measured in the contact area with the tested gasket. The dominant pattern of the characteristic peak, which appears most frequently and varies slightly in shape, can be clearly seen. A maximum peak can also be distinguished.

### 3.3. Analytical Calculations

**Figure 24:**
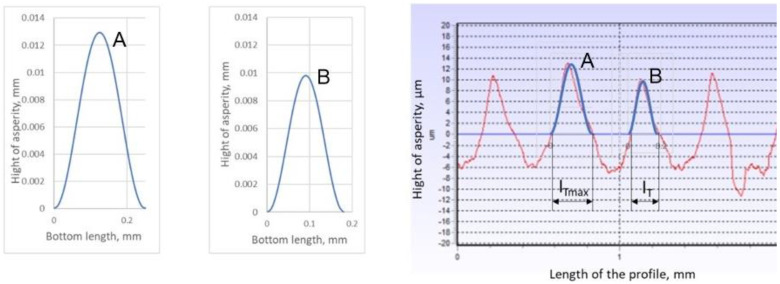
Fragment of the roughness profile of the lower plate of a hydraulic press with the characteristic roughness peaks modelled and plotted.

Figure 24 shows a section of the roughness profile of the lower plate of the hydraulic press, together with the characteristic roughness peaks modelled and plotted. The fit of the peaks was obtained using the method of successive approximations by changing the value of the fractal dimension *D_f_*. The value of *G* was assumed to be the same as in [14]. For a comparable roughness parameter, *Ra* = 4.2 μm, *G* = 1.9 × 10^−8^.

**Figure 25:**
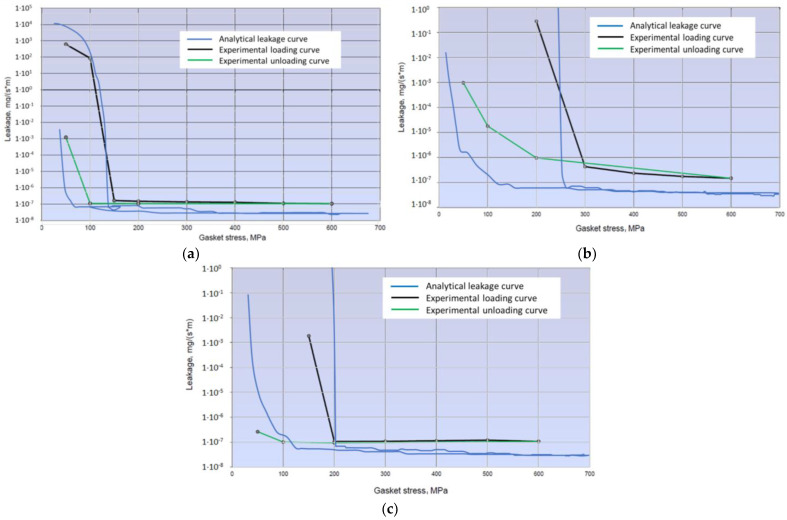
Leakage characteristics obtained from analytical calculations at (**a**) 20 °C; (**b**) 200 °C; (**c**) 400 °C.

Using Equations (5)–(20), the basic parameters describing the permeability and mass leakage of helium through the porous bed formed by the contact between the sealing ridge surface and the hydraulic press plates were determined. The basic components of the analytical model were the parameters determined (from part of the experimental studies) describing the profile of the gasket ridge roughness of the sealed plates. Based on these, the shape of the model fragment of the roughness profile, and its changing cross-sectional area resulting from the gradual penetration of the sealing ridge, was mathematically reflected. Determined on the basis of numerical tests, the degree of depression of the sealing ridge *z* allowed for the following to be calculated: the degree of porosity (according to Equation (16)), the maximum pore diameter (according to Equation (17)), the degree of capillary dislocation (according to Equation (19)), and the fractal dimension of the capillary (according to Equation (18)). These data were the components used for the determination of the permeability described by Equation (5) and its value, in conjunction with the thermodynamic parameters of the gas (viscosity and density of helium at a given temperature) and the numerically calculated contact width (determining the inner and outer radius of the contact surface of the inner sealing ridge), allowing for the calculation of the mass leakage from Equation (20). The aforementioned parameters are shown in fragments in Table 2. The graphical progression of the analytical characteristics, as a function of temperature, on the background of the experimental characteristics, is shown in Figure 25. Helium parameters as a function of temperature are shown in Table 3.

From the course of the characteristics shown in Figure 25, it can be seen that the proposed mathematical model represents the real phenomenon well. This is evidenced by their similarity to the characteristics obtained experimentally (Figure 22).

The hysteresis that developed between the load and unload curves is the result of the plastic deformation of the inner ridge. This is well illustrated by the graphs in Figure 20, which show the variation in the internal contact area of the sealing ridge as a function of its deformation. In addition, the undulation of the model characteristic curve is mainly due to the stepwise variation in the contact area obtained from the numerical calculations.

What is noteworthy here is the explanation for achieving the minimum leakage value. Certain constraints had to be introduced into the mathematical model, resulting from the minimum value of the degree of porosity and the minimum pore diameter. Without their introduction, an increase in the cavity of the sealing ridge inside the model pore would have caused these parameters to gradually move towards 0.

**Figure 26:**
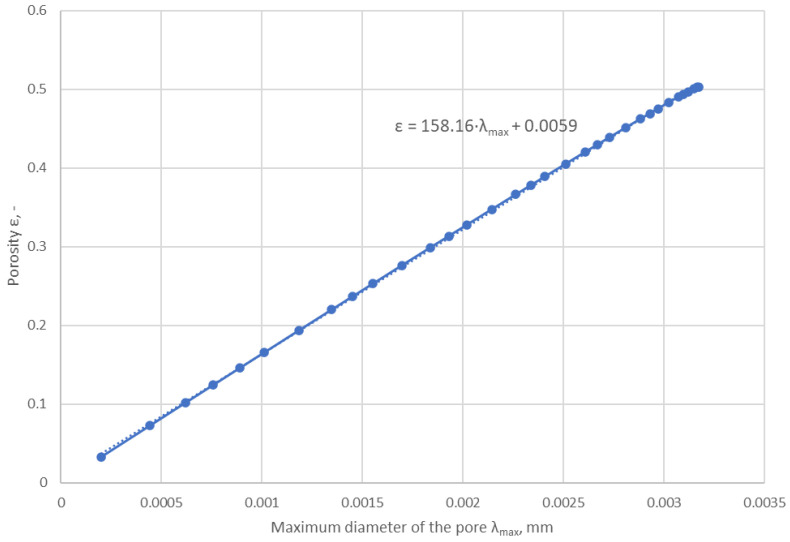
Variation in the degree of porosity as a function of the maximum pore diameter.

The first constraint was introduced on the basis of the minimum dimension of the pore λ*_min_* that could be inserted into the area of the square whose sides form the distances between neighbouring atoms of the metallographic lattice. A value of two angstroms was taken as this dimension, i.e., a value equal to 2 × 10^−7^ mm. By plotting the degree of porosity calculated by Equation (16) as a function of the maximum pore diameter λ*_Pmax_*(*z*), it was possible to determine the critical porosity at which the maximum pore diameter was λ*_max_* = 100·λ*_min_* = 2 × 10^−5^ mm (see Figure 26). For the value λ*_max_* = 2 × 10^−5^ mm, the minimum value of the degree of porosity ε = 0.00906 was determined by means of the approximation formula outlined in Figure 26.

Another constraint was the introduction of a value for the fractal dimension, which also tends towards 0 as the degree of porosity decreases. In the case of the two-dimensional model, the value of this parameter ranges from 2 to 1, i.e., the lower value was set at 1. It should also be noted that the effect of increased temperature necessitated the introduction of a certain correction, namely the correction of the average pore diameter, which resulted from thermal expansion. This correction was made using the following equation:

(24) $$\lambda_{maxT}=\lambda_{max}\cdot \Delta T\cdot \alpha_{C}$$

where λ*_maxT_*—maximum pore diameter at elevated temperature; Δ*T*—temperature increase of more than 20 °C; and α*_C_*—coefficient of linear expansion of the hydraulic press plates material.

It should be noted that the proposed analytic model predictions and the experimental leakage curves (Figure 25) have not been checked for accuracy and consistency since the gasket compression test result from only one gasket was considered (Figure 21). Furthermore, the proposed analytical model should be checked in regard to the sensitivity of the parameters that have an influence on the permeability and the leakage rates. This will be the topic of future research work.

## 4. Discussion

The numerical calculations of the gasket, carried out in the first part of this paper, allowed for the determination of the basic data related to its deformation, i.e., the deformation of the ridges, the measurement of the contact area of the ridges as a function of its compression, and the determination of the average contact stress related to the base of the three ridges of the gasket. These results, particularly in relation to the inner ridge of the gasket, provided the necessary input to the analytical model. It is the inner ridge of the gasket that is responsible for its tightness as it represents the first leakage restriction threshold in the fluid flow path through the gasket. From the compression characteristics obtained from the numerical calculations, shown in Figure 18, the compression level of the gasket under a pressure of 700 MPa, depending on set temperatures of 20 °C, 200 °C, and 400 °C, is 11.8%, 16.3%, and 17.4%, respectively. The contact area of the inner ridge at the maximum compression level was 30 mm^2^ at 20 °C and about 60 mm^2^ at 200 °C and 400 °C. The maximum deformation of the inner ridge was 0.175 mm, 0.21 mm, and 0.25 mm for 20 °C, 200 °C, and 400 °C, respectively, which represented 34%, 42%, and 50% of the initial ridge height.

The experimental tests carried out in the second part of this paper allowed for the determination of the actual compression characteristics. This made it possible to verify the correctness of the numerical model assumptions. As it turned out, the course of the actual characteristics against the numerically determined characteristics differed only slightly from each other. The largest deviations occurred in the initial compression phase of the gasket. These deviations were probably due to slight differences between the actual dimensions of the gasket ridges and the geometric model used in the calculations. The clearances between the test stand components and the measurement accuracy of the force and displacement sensors probably also had an additional influence.

From the leakage characteristics obtained, it can be observed that the leakage decreases very intensively as the contact pressure increases. In the pressure range from 50 MPa to about 200 MPa, the leakage changes by up to four orders of magnitude. At a certain contact pressure limit, a minimum leakage of approx. 1 ×10^−7^ mg/(m·s) is reached depending on the temperature used in the tests. Further increases in contact pressure beyond the value determining the minimum leakage no longer cause significant changes. During the unloading phase of the gasket, i.e., the reduction in contact pressure, the minimum leakage point shifts towards lower contact pressure values. This is a result of the permanent deformation of the inner ridge of the gasket, its increased contact area, and its better alignment with the surface irregularities of the hydraulic press plates, providing better resistance to the passage of fluid.

As shown by the measurements of the roughness profile of the press plates, its shape changes in a regular manner, in which the profile’s characteristic peak and maximum peak can be distinguished. The characteristic peak reaches a height of about 0.0105 mm and a base length of 0.181 mm, while the height and base width of the maximum peak are 0.013 mm and 0.248 mm, respectively.

The analytical calculations carried out in the final stage of the paper showed that the analytical model proposed in the theoretical part very well represents the phenomenon of fluid flow at the interface between the sealing edges and the sealed surfaces. The model describing the permeability of the porous bed, adopted from the literature, in conjunction with Darcy’s model of flow and the geometric model proposed by the authors, which determines the variable (under pressure) geometry of the pore through which the flow occurs, reflects the real phenomenon very well.

This is evidenced by the leakage characteristics plotted from analytical and numerical calculations as a function of gasket contact stress. Both their course and the characteristic point at which the transition occurs to reach minimum leakage values are very close to the actual characteristics determined at a given temperature. The analytical model required the introduction of certain constraints. These were the application of a minimum degree of porosity and a limit on the maximum pore diameter. Without these constraints, the leakage rate would have tended towards 0 with increasing pressure. The limiting value for the maximum pore diameter was determined from the minimum pore diameter that would be inserted into the region formed between adjacent atoms of the metal structure. This value was assumed to be 2 × 10^−7^ mm, i.e., 2 angstroms, and the ratio of minimum to maximum pore diameter was determined from Equation (5). By plotting the relationship between the analytically calculated degree of porosity and pore diameter, the minimum value of the degree of porosity was determined.

## 5. Conclusions

The following conclusions can be drawn from the numerical and analytical calculations and experimental studies carried out in this paper:

1. The numerical model proposed in this paper, reproducing the geometry of a sharp-edged gasket located between two parallel plates of a hydraulic press, allows for the degree of deformation of the gasket to be modelled in a sufficiently accurate manner, both at room temperature and at an elevated temperature;
2. The gasket presented in this paper is characterised by a very low leakage rate. Its minimum value is 1 × 10^−7^ mg/(s·m) and is achieved at a pressure of approximately 200 MPa;
3. Loading the gasket outside the pressure area determining the minimum leakage causes the critical leakage value to shift to the side of the lower contact pressure during the gasket unloading phase;
4. As the temperature increases during the gasket test, the stress unloading curve becomes smoother. This means that an increase in temperature maintains a higher gasket tightness;
5. The level of deformation of the inner ridge of the gasket determines its tightness, while the other ridges act as secondary barriers;
6. The analytical calculations show that the achievement of minimum leakage is related to the complete filling of a section of the roughness profile by the sealing ridge of the model channel, which establishes the limiting minimum pore diameter;
7. The shape of the leakage characteristics, in the unloading phase, is determined by the level of deformation of the ridge, particularly its contact width resulting from permanent plastic deformation;
8. A further perspective for this work is to consider the non-uniform stress distribution on the gasket ridges that occurs in a real bolted flange joint;
9. The proposed analytical model should be checked for the sensitivity of the parameters affecting the permeability and leakage rates.

## Figures and Tables

**Table 1:** Mechanical properties of the materials adopted in the model [36,37].

Part	Material	*T*, °C	*E*, MPa	ν, No Unit	*E_u_*, MPa	*R_p_*_0.2_, MPa	α_c_, 1/°C × 10^−6^
Gasket	800HT	20	94,000	0.32	1400	453	15.6
		200	85,000	0.34	1192	430	15.9
		400	75,000	0.37	962	404	16.3
Plates	Inconel^®^ Alloy 625	20	207,000	0.28	N/A	N/A	12.8
		200	197,000	0.29	N/A	N/A	13.1
		400	188,000	0.30	N/A	N/A	13.6

**Table 2:** Summary of selected results related to the calculation of permeability and mass leakage at 20 °C.

*A_pmax_*(*z*), mm^2^	ε, No Unit	λ*_max_*, mm	*D_f_*, No Unit	τ, No Unit	*K_V_*, m^2^	*Q_m_*, mg/(m·s)	*A_IR_*(*z*), mm^2^	*z*, mm	σ, MPa
0.000865	0.503	0.003173	1.850	1.455	8.562 × 10^−16^	24,378.6	3.205	0.0007	16.2
0.000864	0.502	0.003166	1.850	1.456	8.476 × 10^−16^	24,213.9	3.145	0.0016	20.7
0.000861	0.500	0.003148	1.849	1.459	8.261 × 10^−16^	23,880.5	3.066	0.0029	25.2
0.000855	0.496	0.003122	1.848	1.465	7.945 × 10^−16^	23,575.0	3.000	0.0043	28.6
0.000849	0.493	0.003099	1.846	1.471	7.674 × 10^−16^	22,883.7	2.967	0.0053	30.9

**Table 3:** Properties of helium as a function of temperature.

Property	Temperature		
	20 °C	200 °C	400 °C
Density, kg/m^3^	0.1634	0.1025	0.0708
Dynamic viscosity, Pa·s	1.70 × 10^−5^	2.73 × 10^−5^	3.48 × 10^−5^

## Data Availability

The original contributions presented in the study are included in the article, further inquiries can be directed to the corresponding author.

## Nomenclature

**Symbol**	**Description**	**Unit**
*A_IR_*(*z*)	Contact area of the inner sealing ridge under deformation	mm^2^
*A_Pmax_*(*z*)	Area of the maximal pore as a function of ridge immersion	mm^2^
*A_Tmax_*, *A_Vmax_*	Maximum cross-sectional areas of the top peak and the valley peak, respectively	mm^2^
*b*(*z*)	Contact width of the inner sealing ridge	mm
***B***_0_	Linear strain–displacement transformation matrix	no unit
***B*** *_L_*	Linear strain–displacement transformation matrix, which depends on the displacement	no unit
***B*** *_N_*	Nonlinear strain–displacement transformation matrix	no unit
*D*, *D_f_*	Fractal dimension and fractal dimension of tortuous capillaries, respectively	no unit
***D***	Elasticity matrix	no unit
*E*, *E_u_*	Young modulus, elastic, and tangent in plastic zone, respectively	MPa
*G*	Fractal roughness constant	no unit
*h*	Height of the porous structure	mm
***K***_0_	Small displacement stiffness matrix	no unit
***K*** *_d_*	Large displacement stiffness matrix	no unit
***K*** *_T_*	Tangent stiffness matrix	no unit
*K_V_*	Permeability	mm^2^
***K*** *_σ_*	Initial stress matrix dependent on the stress level	no unit
*l_Tmax_*, *l_Vmax_*	Base lengths of the maximum top peak and valley peak, respectively	mm
*L*	Sample length	mm
*n*	Frequency index of asperities	no unit
*M*	Number of superposed ridges	no unit
*p_o_*, *p_t_*	Pressure inside and outside of the cylinder, respectively	MPa
*P_Pmax_*(*z*)	Perimeter of the pore as a function of ridge immersion	mm
*P_Tmax_*, *P_Vmax_*	Perimeters of the maximum top peak and valley peak, respectively	mm
*Q_m_*	Mass flow per perimeter	mg/(s·m)
*r_i_*, *r_o_*, *r_IRi_*, *r_IRo_*, *r_IRm_*, *r_AV_*	Radius, inner, outer, and inner of the inner ridge, outer of the inner ridge, mean of the inner ridge, and average radius of the cylinder, respectively	mm
*R_p_*_02_	Yield strength	MPa
*S*	Cross-section of the flow	mm^2^
***S***	Piola–Kirchhoff second stress tensor	no unit
*T*	Temperature	°C
*x*, *y*, *z*	Coordinates in vertical and horizontal positions, respectively	mm
α	Opening angle of the sealing ridge	°
α*_c_*	Coefficient of the thermal expansion	1/°C
δ*_Tmax_*, δ*_Vmax_*	Maximum height of the top peak and valley peak, respectively	mm
Δ***F***	Difference between applied force and resistance force (vector)	no unit
Δ***U***	Generalised displacement vector	no unit
ε(*z*)	Porosity level as a function of ridge immersion	no unit
η	Dynamic viscosity	Pa·s
λ_max_, λ_min_	Diameter of the pore, maximum and minimum, respectively	mm
ν	Poisson ratio	no unit
ρ	Fluid density	kg/m^3^
γ	Frequency spectrum	no unit
τ	Tortuosity of the capillaries	no unit
$\varphi_{a}$	Random number	no unit
